# Supplementary material for: Identification and Characterization of a Novel Endo-β-1,4-Xylanase from Streptomyces sp. T7 and Its Application in Xylo-Oligosaccharide Production
Source: Molecules. 2022 Apr 13;27(8):2516. doi: 10.3390/molecules27082516 (PMC9032680; doi:10.3390/molecules27082516)
Supplement: Supplementary file 1 [file molecules-27-02516-s001.zip › molecules-1618562-supplementary.pdf]

Identification and Characterization of a Novel Endo-β-1,4-Xylanase from *Streptomyces* sp. T7 and its Application in Xylo-oligosaccharide Production

School of Biological Science and Biotechnology, University of Jinan, China

*Corresponding author. Tel: +86 531 82766825; fax: +86 531 82765807

E-mail: bio_huangzs@ujn.edu.cn

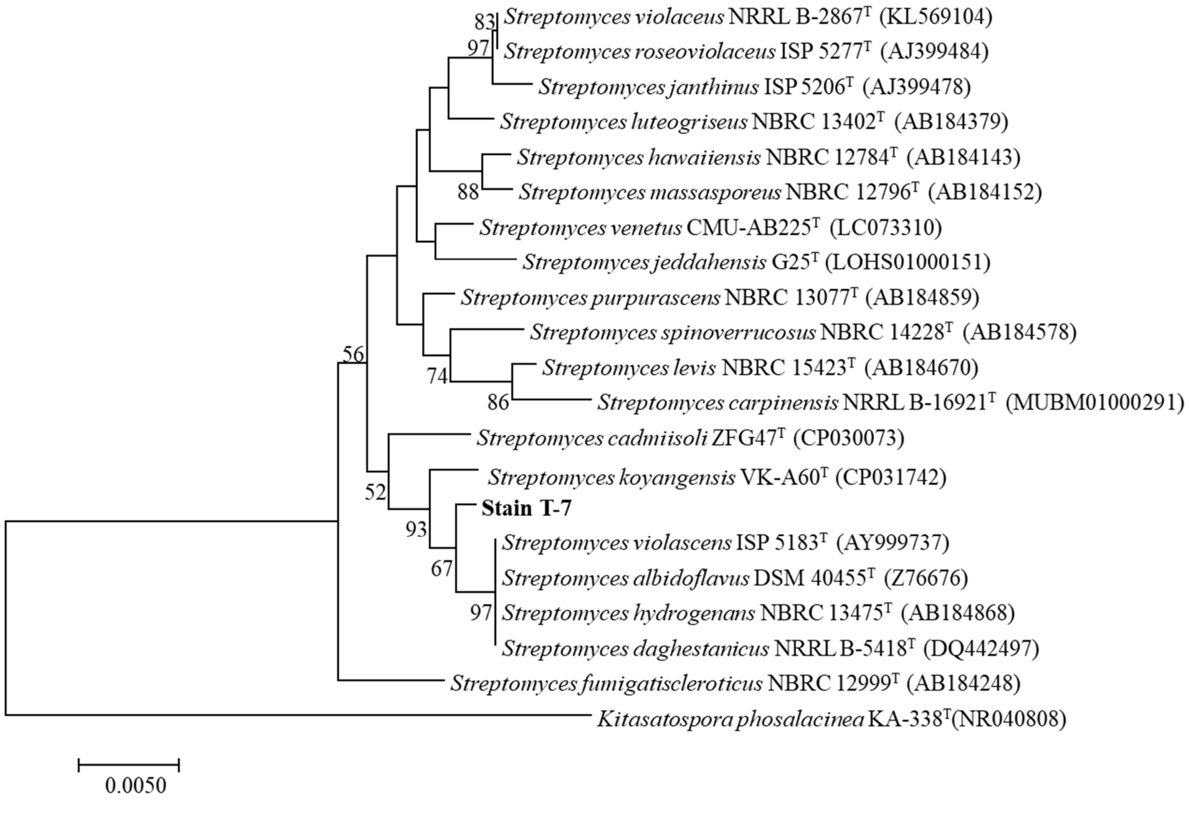

Figure S1. Neighbour-joining phylogenetic tree based on 16S rRNA gene sequences showing the position of strain T7 among its phylogenetic neighbours. Kitasatospora phosalacinea KA-338^{T} was used as outgroup. Bootstrap values (>50 %) based on 1000 replications are shown at branch nodes. Bar, 0.005 substitutions per nucleotide position.

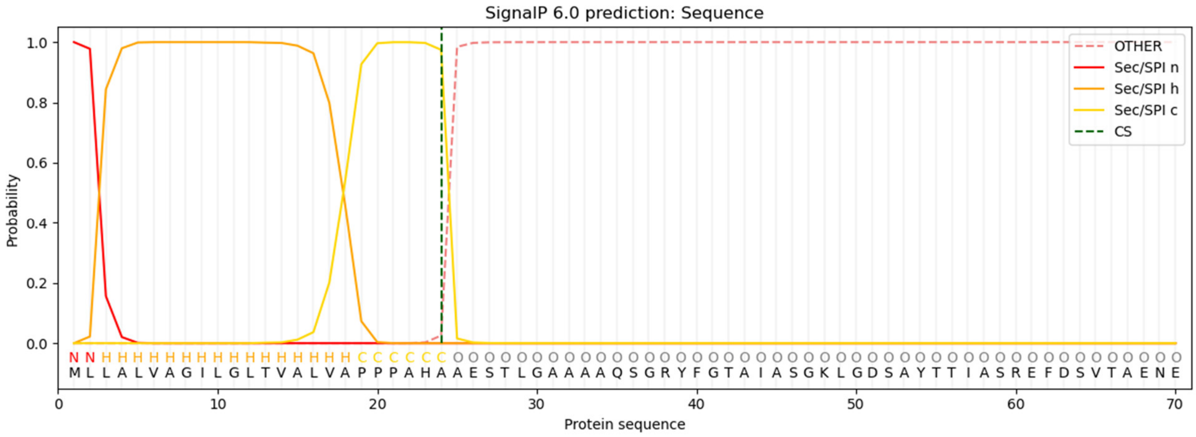

Figure S2. Predictive signal peptide sequence of xynST7

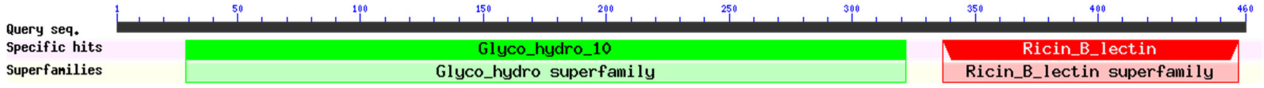

Figure S3. Conserved domains of xynST7

Amino acid composition:

Ala	(A)	46	10.6%
Arg	(R)	22	5.1%
Asn	(N)	25	5.7%
Asp	(D)	30	6.9%
Cys	(C)	10	2.3%
Gln	(Q)	26	6.0%
Glu	(E)	9	2.1%
Gly	(G)	52	12.0%
His	(H)	5	1.1%
Ile	(I)	14	3.2%
Leu	(L)	26	6.0%
Lys	(K)	14	3.2%
Met	(M)	6	1.4%
Phe	(F)	12	2.8%
Pro	(P)	12	2.8%
Ser	(S)	36	8.3%
Thr	(T)	35	8.0%
Trp	(W)	14	3.2%
Tyr	(Y)	14	3.2%
Val	(V)	27	6.2%
Pyl	(O)	0	0.0%
Sec	(U)	0	0.0%

Figure S4. Total amino acid composition (%) of xynST7. Total numbers of negatively (Asp + Glu) and positively (Arg + Lys) charged residues were 39 and 36, respectively.

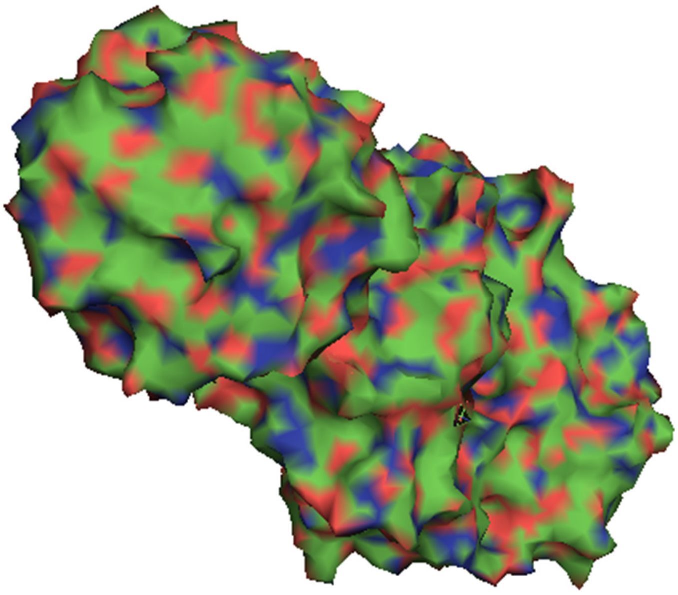

Figure S5. Surface representation of xynST7. Negatively and positively charged surfaces are coloured red and blue, respectively.
